# The role of cancer-associated fibroblasts in esophageal cancer

**DOI:** 10.1186/s12967-016-0788-x

**Published:** 2016-01-29

**Authors:** Jiangfeng Wang, Guangyu Zhang, Jianbo Wang, Lu Wang, Xiaochen Huang, Yufeng Cheng

**Affiliations:** Department of Radiation Oncology, Qilu Hospital of Shandong University, No 107 West Wenhua Road, Jinan, 250012 People’s Republic of China

**Keywords:** Esophageal cancer, Cancer-associated fibroblasts, Carcinogenesis, Progression, Metastasis, Prognosis, Treatment

## Abstract

Fibroblasts are known as critical stromal cells in wound healing by synthesizing extracellular matrix and collagen. A subpopulation of them is called cancer-associated fibroblasts (CAFs), because their production of proteins participated in various biological activities including tumor cell proliferation, invasion and metastasis. Currently some studies shed light on their role in esophageal cancer which was an aggressive cancer with a dismal survival and high rate of metastasis. Thus, to find cures for it relies on elucidating the epithelial-fibroblasts crosstalk. Herein, we reviewed the present knowledge of the CAFs’ role in esophageal premalignant condition, cancer initiation, progression, metastasis and prognosis prediction and further provided some insights into its clinical application.

## Background

More than 450,000 people worldwide are inflicted with esophageal cancer featuring early metastatic spread and poor prognosis every year, whose overall 5-year survival ranged between 15 and 25 % [1]. The poor outcomes are not only related to the tumor-cell but also impacted by the stromal fibroblasts [2]. Previously, the investigations of fibroblasts usually focused on wounds healing. Activated fibroblasts with high-proliferation and high-secretion phenotype generate extracellular matrix (ECM) and regulate inflammation. Once the wounds are healed, the number of activated fibroblasts is greatly reduced and the quiescent fibroblasts are restored [3]. Currently, some studies concentrated on the role of perpetually activated fibroblasts in the microenvironment of various types of cancers including esophageal cancer [3, 4]. And these activated fibroblasts are known as cancer-associated fibroblasts (CAFs).

It becomes clear that CAFs are prominent regulators involved in the entire course of cancer development. They provide esophageal cancer cells with a suitable environment for carcinogenesis, proliferation, angiogenesis and invasion via secreting factors [2, 5–10]. Thus, the aims of the study were to summarize CAFs’ role in esophageal cancer and to provide some insights into its future clinical application.

### The origins of CAFs

**Fig. 1:**
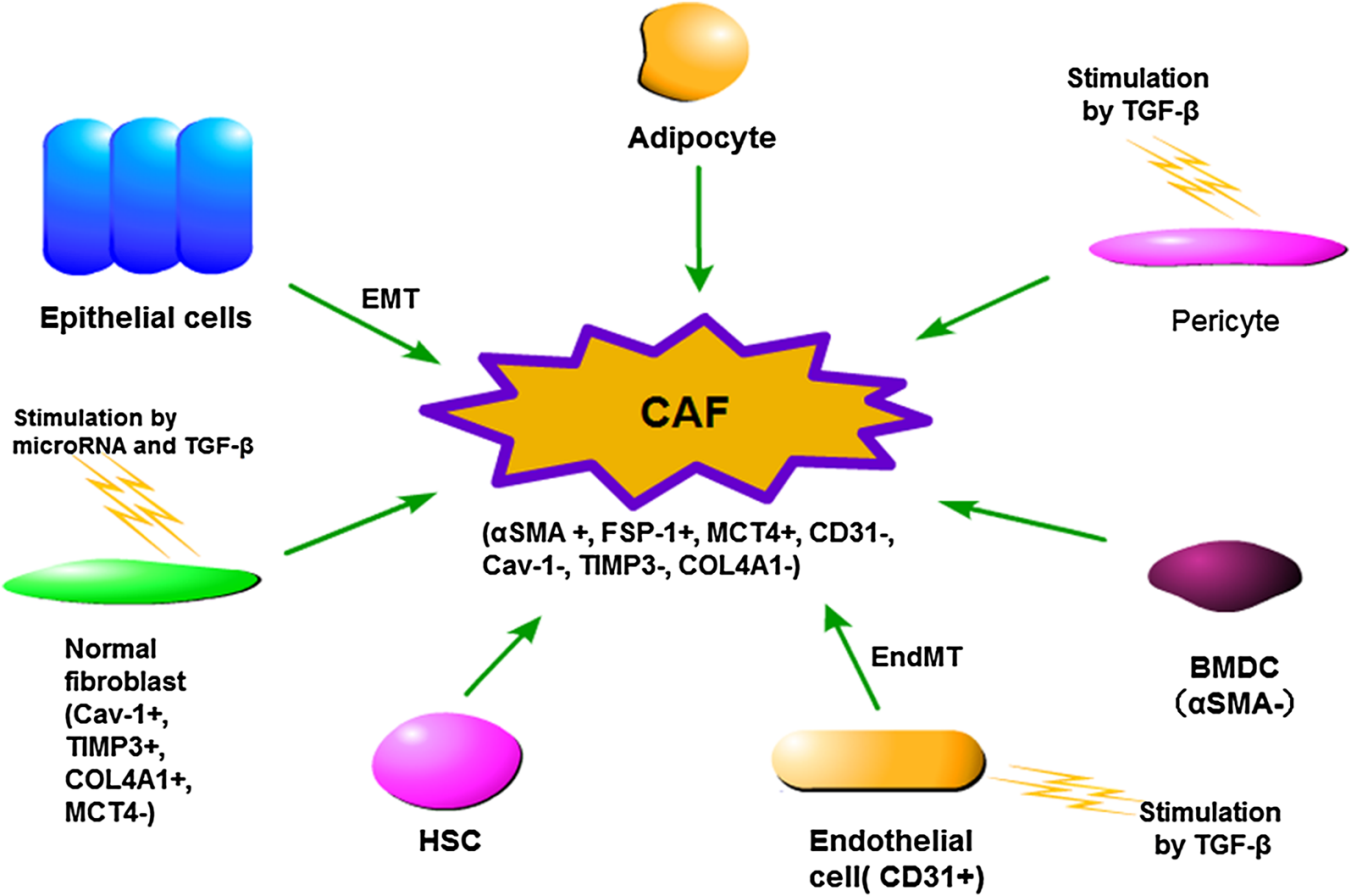
Origin of CAFs. CAFs can derive from a variety of cells, including normal fibroblasts (NFs), bone marrow-derived cells (BMDCs), endothelial cells, hematopoietic stem cells (HSCs), epithelial cells, pericytes and adipocytes. Previerous studies showed their nature to transform to CAF phenotype. TGF-β and microRNAs were reported as the drivers of this transformation

To better understand the impact of CAFs on esophageal cancer development, the first step is to know their diverse origins, which included normal fibroblasts (NFs), bone marrow-derived cells (BMDCs), endothelial cells, hematopoietic stem cells (HSCs), epithelial cells, pericytes and adipocytes [11] (Fig. 1).

NFs in esophageal cancer were reported to be a source of CAFs in response to the paracrine of cancer cells. Nouraee et al. [12] co-cultured NFs with esophageal carcinoma cells and observed CAF markers were up-regulated. It was suggested that some microRNAs may got involved in this process. The transformation may be attributed to the elevated release of microRNA-21 from esophageal squamous cell carcinoma cells (KYSE-30). In addition, microRNA-27 was also observed to manipulate the transformation of NFs into CAFs [13]. Transforming growth factor beta (TGFβ) also was promoter of this process [14]. BMDCs were another source of esophageal activated fibroblasts. Hutchinson et al. [15] transplanted mice with bone marrow expressing beta galactosidase. Then the mice suffered Barrett’s metaplasia, in which authors found activated fibroblasts labeled with not only their specific marker α smooth muscle actin (SMA), but also beta galactosidase. It implicated they were derived from BMDCs. In vivo, authors found BMDCs-derived CAFs in a patient with esophageal adenocarcinoma (EAC) [15]. In this research, a male patient was diagnosed as EAC 10 years after bone marrow transplantation from a female donor. Through X/Y fluorescent in situ hybridization and anti-α-SMA staining, they discovered 12.57 % CAFs were originated from the donor bone marrow [15]. However, the small sample size was the limitation of this study. It should be verified in more patients. In other cancers, endothelial cells, HSCs, epithelial cells, pericytes and adipocytes were proved as CAFs resources [11, 16–20], but they remained unclear in esophageal cancer, which needed further researches.

### The role of fibroblasts in esophageal premalignant condition

Reflux esophagitis is stimulated by gastric acid and bile reflux. Chronic exposure to gastroesophageal reflux will transform esophageal squamous epithelium to columnar epithelium, which is known as Barrett’s esophagus (BE). It is the precancerous lesion of esophageal cancer [21]. Therefore, in order to investigate the whole course of cancer development, some studies focused on the relationship between fibroblasts and precancerous disease.

Interleukin (IL)-1β and IL-6 were found in mucosal specimens of reflux esophagitis patients. In the study of Rieder et al. [22], treatment of IL-1β on human esophageal fibroblasts induced increased production of IL-6 which was the critical proinflammatory cytokines.

BE metaplasia could be induced by fibroblasts-derived factors. In esophageal mucosal biopsy specimens from reflux esophagitis patients, Farzana et al. [5] found that heparin-binding EGF-like factor (HB-EGF) was robustly expressed in fibroblasts. Additionally, in vitro stimulation of normal esophageal squamous epithelial cell with HB-EGF enhanced expression of the malignant markers including Cdx2, cytokeratin 7 and villin. Cdx2 was involved in intestinal metaplasia [23, 24]. Cytokeratin 7 and villin were columnar cells markers. Proliferation of BE was also promoted by fibroblasts. In Barrett’s esophageal fibroblast culture, the crucial enzyme of prostaglandins E2 (PGE2) cyclooxygenase (COX)-2 was detected. Adding COX-2 inhibitor into epithelial cell cultures suppressed its proliferation, while PGE2 treatment rescued the Barrett’s esophageal epithelial cell proliferation [25]. Taken together, proteins from fibroblasts were responsible for BE’s inducement and proliferation.

### The role of CAFs in esophageal carcinogenesis

Esophageal squamous cell carcinoma (ESCC) initiation is commonly attributed to hot drinks and alcohol intake [26]. Whereas, EAC carcinogenesis is reported to closely correlate to BE [25]. Many factors participating in carcinogenesis were investigated. And CAFs was just one of them.

In ESCC tissues, transforming growth factor β1 (TGFβ1) and hepatocyte growth factor (HGF) were stained in fibroblasts. Their levels were increased gradually from low-and high-grade intraepithelial neoplasia to carcinoma in situ and ESCC groups [27]. So fibroblasts were speculated to promote carcinogenesis of ESCC through TGFβ1 and HGF. However, no further studies investigated the mechanism.

Inflammatory factors produced by CAFs got involved in ESCC carcinogenesis as well. Achyut et al. [28] deleted TGFβ receptor 2 (TβRII) stromal fibroblasts in the mice forestomach. As a result, adjacent epithelial cells showed little expression of cyclin-dependent kinase inhibitor (CKI), high-level COX2, increased proliferation and decreased apoptosis. Then, in human ESCC specimens, they found that TβRII was downregulated in CAFs, inflammatory factors COX2 and DNA damaging indicators were enhanced and CKIs were decreased or even lost. Because squamous cell carcinoma of mouse forestomach was similar to human ESCC, so authors assumed CAFs of ESCC led to DNA damage, unlimited cell proliferation and malignant transformation. However, the limitation of the research is obvious. The cell source of mouse fore stomach led to faint conclusion, which needed robust investigation in ESCC cells.

Verbeek et al. [29] reported that CAF-derived Toll-like receptor-4 (TLR-4) (an innate immune system activator) was involved in the progression from BE to EAC. Expression of TLR-4 progressively increased from reflux esophagitis patients, patients with BE, to those with EAC. It was found to boost COX-2 level in BE,which was critical in EAC carcinogenesis, because it owned anti-apoptotic potential in vitro [30] and its inhibitor had ability to reduce incidence of esophageal cancer in vivo [31]. So authors supposed TLR-4 played its role in neoplastic progression in BE through facilitating COX-2 expression.

### The role of CAFs in the proliferation and angiogenesis of esophageal cancer

Fibroblast growth factor (FGF) and HGF were involved in many biological activities including proliferation, differentiation, and cellular motility and morphogenesis [32, 33]. In ESCC, they could be expressed in CAFs and promoted tumor cells proliferation via HGF/MET and FGF/FGF receptors (FGFR) pathways. Shin et al. [6] observed that ESCC cells proliferation was boosted when they were cultured with FGF and HGF. And this process could be blocked by MET inhibitor and FGFR inhibitor. FGFR2-positive CAFs supported ESCC proliferation by creating a suitable microenvironment. A study conducted by Chunyu et al. [2] compared different expression of 126 genes between CAFs from esophageal tumor tissues and NFs, and revealed that FGFR2 was one of the most differently expressed genes. Cancer cells grew rapidly when cultured in conditioned medium from FGFR2-positive CAFs. The medium was detected to contain growth factors and transcription factors, such as FGF, transcription factor AP-2α and AP-2γ, all of which were suitable for esophageal cancer cell proliferation.

Wingless-type MMTV integration site family member 2 (Wnt2) was a member of Wnt signaling pathways which played multiple roles in regulating cellular behavior. It was revealed that Wnt2 secreted by CAFs was able to mediate tumor cell growth. Fu et al. [34] investigated tumor tissues from 51 primary ESCC patients by immunohistochemical staining and found that Wnt2 was detectable in 82.4 % cases. The majority of Wnt2-positive cells were located around tumor nest. Moreover, Wnt2 and the fibroblast marker vimentin were verified to co-express in the same cells. Thus, the authors considered Wnt2-positive cells as CAFs. It was further demonstrated that the proliferation rate of ESCC cells was higher in conditioned medium containing secreted Wnt2 than that of control group.

Vascular endothelial growth factor (VEGF) could stimulate angiogenesis and anti-VEGF therapies were crucial in some cancer treatments [35]. Noma et al. [14] created a novel 3D model to study angiogenesis in ESCC. It was reported angiogenesis and high-level VEGF existed in the model but not in that without CAFs. To further test the role of CAFs in angiogenesis, the authors suppressed their activation by adding TGFβ inhibitors. As a result, VEGF and vascular network formation were hardly detected. In contrast, both of them were rescued when the suppression was removed. Therefore, CAFs were indispensable in angiogenesis.

Additionally, CAFs were able to accelerate EAC growth. Underwood et al. [9] respectively injected OE33 (EAC cell line), OE33+NFs and OE33+CAFs into three groups of mice. The tumor volume of the mice injected by EAC cells plus CAFs reached 500 mm3 earliest. The trend was verified by another EAC cell line OANC1. Another study [36] revealed that CAFs expressed VEGF to facilitate angiogenesis during tumor growth. This provided evidence for the similar involvement of CAFs in angiogenesis in ESCC and EAC.

### The role of CAFs in esophageal cancer invasion and metastasis

Invasion and metastasis were the most common causes of cancer-related death, which were related to some factors from CAFs including HGF, transforming growth factor beta-induced protein (TGFβI), Wnt2, periostin and podoplanin [7–10].

HGF was an invasiveness-promoting factor in ESCC. This was supported by Grugan et al. [10] who revealed invasive epithelial cells were induced by the stimulation of CAF-derived HGF. And the process could be blocked by MET (known as the receptor of HGF) inhibitor and be enhanced by MET activation.

TGFβI exhibited different role in cancer progression. For example, it promoted colon cancer cells metastasis and suppressed mesothelioma progression [37, 38]. In ESCC, TGFβI was a promoter which was mainly detected in fibroblasts. Authors verified it by wound healing and invasion assays. Compared with control group, migration and invasion abilities of ESCC cells were suppressed dramatically when they were cocultured with TGFβI-downregulated fibroblasts [8].

Epithelial-mesenchymal transition (EMT) was a process in which the epithelium-origin tumor cells lost the polarity and intercellular adhesion, and gained a propensity for migration and invasion to become mesenchymal cells. CAF-derived Wnt2 could enhance motility and invasiveness of esophageal cancer cells via EMT. Incubated in medium containing Wnt2, ESCC cells showed down-regulated epithelial markers, up-regulated mesenchymal markers and enhanced capacity to invade through extracellular matrix by up to 75 % [34].

In terms of EAC cells invasiveness, Underwood et al. [9] demonstrated two EAC cell lines FLO-1 and OE33 showed a more than twofold increasing invasion in Transwell invasion assays when exposed in conditioned medium from CAFs compared with that from NFs. This trend was in accordance with results of the organic culture. They measured the average tumor invasion depth, the number and area of invading tumor islands as the parameters of cancer cells invasiveness. Strengthened invasion of FLO-1 and OE33 cells was observed in CAFs-containing gels. In order to clarify its mechanism, authors examined the CAFs and NFs conditioned medium and found more periostin in the former one. Periostin is crucial for cell adhesion and migration. Then, they respectively eliminated periostin in RNA and protein level from two groups of CAFs-conditioned medium. Neither did the medium support EAC invasion in Transwell assay nor in the organic culture. This was restored through periostin addition. In addition, authors found PI3k–Akt pathway activated by periostin was involved in the process. This supported the notion that EAC invasiveness was fueled by periostin secretion by CAFs. Except for periostin, the lymphatic endothelium marker podoplanin expressed in CAFs correlated with the EAC lymphovascular invasion and lymph node metastasis [39].

A majority of studies merely focused on ESCC or EAC alone, but there were a few studies investigating proteins in both of them, showing the histological-specific feature. For example, the stromal podoplanin and carbonic anhydrase IX (CA IX) which were associated with lymph node metastasis in EAC did not make the same impact on ESCC [39–41]. Notably, the association of CA IX with CAFs was verified in EAC rather than ESCC [41]. Further study should clarify its source in ESCC.

### CAFs and esophageal cancer prognosis

As a highly lethal disease, it is significant to identify prognosis indicators of esophageal cancer, which impacts the treatment options. Besides their role in carcinogenesis, proliferation, angiogenesis, invasion and metastasis, proteins of CAFs were reported as prognosis markers for esophageal cancer patients. Studies revealed that immature CAF phenotype and CAF indicator α-SMA was respectively related to the poor survival of ESCC and EAC patients [9, 42].

In ESCC patients, CD10 was expressed in CAFs. Authors found the CD10 overexpressing was correlated with decreased tumor differentiation, poor overall survival (OS) and disease free survival (DFS) [43]. Fu et al. [34] examined 51 tumor specimens from ESCC patients who received operation alone without preoperative treatment. CAFs-derived Wnt2 showed significant association with lymph node metastasis and short median survival time (Wnt2-positive ESCC vs Wnt2-negative ESCC, 16 vs. 51 months). Ozawa et al. [8] observed strong expression of TGFβI in fibroblasts. Its expression was an independent predictor of OS. Thus, all the literatures reviewed above led to the conclusion that some productions of CAFs were a potential prognostic marker in ESCC.

In EAC patients, CAF-originated periostin also served as a survival predictor. Periostin was detected where there was high α-SMA expression. This suggested that periostin was released from CAFs. Further study demonstrated that patients with periostin-positive in tumor had worse prognosis than those periostin-negative patients in OS (46.80 vs. 76.45 months) and DFS (47.45 vs. 80.67 months), respectively [9]. The transmembrane sialoglycoprotein podoplanin exhibited its prognostic value in EAC patients as well. Schoppmann et al. [39] investigated EAC patients who underwent operation and found that podoplanin-expressing CAFs were positively associated short OS (42 vs. 105 months) and mean DFS (42 vs. 89 months). So no matter what disease patients suffered, high level of these proteins predicted poor prognosis.

### Future clinical application of CAFs in esophageal cancer treatment

Despite comprehensive treatment, the prognosis of esophageal cancer remains dismal. Present therapies such as chemotherapy, radiotherapy and targeted-treatment emphasized elimination cancer cells per se. However, according to the present review, carcinogenesis, cancer angiogenesis, metastasis and prognosis were all remarkably affected by stromal microenvironment. Thus, we believed blocking epithelial-fibroblasts crosstalk may be a promising therapeutic management of esophageal cancer.

Currently, studies concerning clinical CAFs-targeted treatment were limited to in vitro studies and animal models. In the study of Shin et al. [6], ESCC cells proliferation was increased when they were incubated in CAFs supernatants. This could be impaired by MET inhibitor PHA-665752 and FGFR inhibitor PD-173074. Achyut et al. [28] deleted TβRII in fibroblasts. They found inflammation and squamous cell carcinoma developed in the forestomach of the mouse. When mouse were treated with anti-inflammation celecoxib, the tumor mass was decreased and their life expectancy increased from 28 to 49 days. The authors believed this model in forestomach could represent ESCC due to similar histology and functional behavior. Although studies on MET inhibitor, FGFR inhibitor and anti-inflammation treatment in cancer suppression were limited, they may provide a potential treatment method for further investigation.

Precisely predicting prognosis of esophageal cancer had long been a focus in clinical practice, for it may affect treatment choice. As was mentioned above, some factors from CAFs may serve as potential biomarkers for prognostic prediction, though few of them were used in clinic by far. Obstacles for its clinical use may be that the detection process is time-consuming and costly. Thus, further investigation was needed.

## Conclusion

CAFs are a subtype of activated fibroblast and originate from numerous types of cells. They have pleiotropic functions in esophageal carcinogenesis, proliferation, angiogenesis, metastasis and prognosis prediction. We reviewed CAFs sources and summarized the role of CAFs-derived proteins in each step of esophageal cancer development, the details of which were shown in Table 1. Podoplanin and CA IX exerted different impact on ESCC and EAC, while VEGF exerted same impact. Given studies elucidating interaction between CAFs and tumor cells, many clinical investigations were conducted. It was commonly suggested factors derived from CAFs served as negative prognosis predictor. Additionally, a few explorations of preclinical treatment targeting the proteins have already been made. Though further researches were needed, we believe interpretation of CAFs in esophageal cancer will increase our knowledge of esophageal cancer and favor future clinical practice.

**Table 1:** The role cancer-associated fibroblasts play in esophageal cancer

Stages during esophageal cancer development	Functional factors expressed by CAFs	In RE/BE/ESCC/EAC	Positive/negative function	Mechanism or postulated mechanism^a^	References
Premalignant condition	IL-6	RE	+	Promoting inflammation	[22]
	HB-EGF	BE	+	Promoting metaplasia	[5]
	COX2	BE	+	Promoting proliferation through PGE	[25]
Carcinogenesis	TGFβ1 and HGF	ESCC	+	Unknown	[27]
	TβRII	ESCC	−	TGF-β signaling pathway	[28]
	TLR-4	EAC	+	COX-2/MAPK pathway^a^	[29]
Proliferation and angiogenesis	FGF	ESCC	+	FGF/FGFR pathways	[6]
	HGF	ESCC	+	HGF/MET pathways	[6]
	FGFR2	ESCC	+	Creating suitable environment for cancer cells proliferation	[2]
	Wnt2	ESCC	+	Wnt/β-catenin signaling pathway	[34]
	VEGF	ESCC	+	Promoting angiogenesis	[14]
	VEGF	EAC	+	Promoting angiogenesis	[36]
Invasion and metastasis	HGF	ESCC	+	HGF/MET signaling pathway	[10]
	TGFβI	ESCC	+	Promoting migration and invasion	[8]
	Wnt2	ESCC	+	Promoting EMT of cancer cells	[34]
	Periostin	EAC	+	PI3k–Akt pathway	[9]

Through secreting factors, CAFs exerted an influence on esophageal cancer development

*RE* reflux esophagitis, *BE* Barrett’s esophagus, *IL* interleukin, *HB-EGF* heparin-binding EGF-like factor, *(COX)-2* cyclooxygenase, *TGFβ1* transforming growth factor β1, *HGF* hepatocyte growth factor, *TβRII* TGFβ receptor 2, *TLR-4* toll-like receptor-4, *FGF* fibroblast growth factors, *FGFR* fibroblast growth factors receptor, *Wnt2* wingless-type MMTV integration site family member 2, *VEGF* vascular endothelial growth factor, *TGFβI* transforming growth factor beta-induced protein, *CA IX* carbonic anhydrase IX, *PGE* prostaglandin E, *MAPK* mitogen-activated protein kinases, *EMT* epithelial-mesenchymal transition

^a^ postulated mechanism

## Abbreviations

BMDCs: bone marrow-derived cells
BE: Barrett’s esophagus
CAFs: cancer-associated fibroblasts
CA IX: carbonic anhydrase IX
CKI: cyclin-dependent kinase inhibitor
COX: cyclooxygenase
DFS: disease free survival
EMT: epithelial-mesenchymal transition
EAC: esophageal adenocarcinoma
ESCC: esophageal squamous cell carcinoma
ECM: extracellular matrix
FGF: fibroblast growth factors
FGFR: FGF receptors
IL: interleukin
HSCs: hematopoietic stem cells
HB-EGF: Heparin-binding EGF-like factor
HGF: hepatocyte growth factor
NFs: normal fibroblasts
OS: overall survival
PGE2: prostaglandins E2
SMA: smooth muscle actin
TβRII: TGFβ receptor 2
TLR-4: toll-like receptor-4
TGFβ1: transforming growth factor β1
TGFβ: transforming growth factor beta
TGFβI: transforming growth factor beta-induced protein
VEGF: vascular endothelial growth factor
Wnt2: wingless-type MMTV integration site family member 2
